# Autoimmunity and cystatin SA 1 deficiency behind chronic mucocutaneous candidiasis in autoimmune polyendocrine syndrome

**DOI:** 10.1186/1479-5876-10-S3-P58

**Published:** 2012-11-28

**Authors:** Emma Lindh, Johan Brännström, Petra Jones, Fredrik Wermeling, Signe Hässler, Corrado Betterle, Ben Zion Garty, Mats Stridsberg, Björn Herrmann, Mikael CI Karlsson, Ola Winqvist

**Affiliations:** 1 Translational Immunology Unit, Dept. of Medicine, Karolinska Institutet, Stockholm, Sweden; 2 Endocrinology Unit, Dept. of Medical and Surgical Sciences, University of Padua, Padua, Italy; 3 Dept. of Pediatrics, Schneider Children's Medical Center of Israel, Tel Aviv University, Israel; 4 Section of Clinical Chemistry, Dept. of Medical Sciences, Uppsala University Hospital, Uppsala, Sweden; 5 Section of Clinical Bacteriology, Dept. of Medical Sciences, Uppsala University Hospital, Uppsala, Sweden

## 

Patients with the monogenic disease autoimmune polyendocrine syndrome type I (APS I) develop autoimmunity against multiple endocrine organs and suffer from chronic mucocutaneous candidiasis (CMC), a paradoxical complication with an unknown mechanism. We report that saliva from APS I patients with CMC was defective in inhibiting growth of *C. albicans in vitro* and had reduced levels of a salivary protein identified as cystatin SA1. In contrast, APS I patients with no CMC expressed salivary cystatin SA1 and could inhibit *C. albicans* to the same extent as healthy controls. We evaluated the anti-fungal activity of cystatin SA1 and found that synthesized full length cystatin SA1 efficiently inhibited growth of *C. albicans in vitro*. Moreover, APS I patients exhibited salivary IgA autoantibodies recognizing myosin-9, a protein expressed in the salivary glands that also produce cystatin SA1, thus linking autoimmunity to cystatin SA1 deficiency and CMC. This data suggests an autoimmune mechanism behind CMC in APS I and provide rationale for evaluating cystatin SA1 in antifungal therapy.

